# Crestal Hydraulic Sinus Lift with Simultaneous Implant Insertion: A Retrospective Case Series

**DOI:** 10.3390/dj13050193

**Published:** 2025-04-28

**Authors:** Francesco Mattia Ceruso, Aurea Immacolata Lumbau, Francesco Pernice, Alessandro Mastroianni, Michele Miranda, Silvio Mario Meloni, Marco Gargari, Marco Tallarico, Milena Pisano

**Affiliations:** 1 Department of Dentistry “Fra G.B. Orsenigo—Ospedale San Pietro F.B.F.”, 00100 Rome, Italy; f.m.ceruso@gmail.com (F.M.C.); francescopernice98@gmail.com (F.P.); alemastroianni11@gmail.com (A.M.); marco.gargari@gmail.com (M.G.); 2 Department of Medicine, Surgery, and Pharmacy, University of Sassari, 07100 Sassari, Italy; smeloni@uniss.it (S.M.M.); mtallarico@uniss.it (M.T.); milenapisano@yahoo.it (M.P.); 3 Department of Clinical Sciences and Translational Medicine, University of Rome “Tor Vergata”, 00100 Rome, Italy; michelemiranda.ptv@gmail.com

**Keywords:** sinus lift, hydraulic sinus lift, bone regeneration

## Abstract

**Objectives**: This retrospective study aimed to evaluate the increase in vertical bone height following sinus lift procedures using the CAS (Crestal Approach Sinus) kit technique in combination with tissue-level implants. Additionally, the quantity of bone between the implant apex and the Schneiderian membrane was assessed to determine the effectiveness and safety of this minimally invasive approach. **Methods**: The study included 15 patients (20 implants) who underwent sinus lift procedures with the CAS kit technique and tissue-level implants in the posterior maxilla between September 2021 and October 2024. Inclusion criteria required a minimum residual bone height (RBH) of 2 mm. Cone-beam computed tomography (CBCT) scans were used for initial screening, and panoramic radiography evaluated outcomes at implant placement and nine months postoperatively. Primary outcomes included implant and prosthetic survival rates, as well as biological and technical complications. Secondary outcomes were vertical bone height and the amount of bone above the implant tip. Statistical analyses were conducted using the Wilcoxon signed-rank test with a significance level of 0.05. **Results**: All implants achieved successful osseointegration, with no implant or prosthetic failures and no biological or technical complications reported. The mean RBH at implant placement was 4.2 ± 1.4 mm, which increased to an overall membrane elevation of 13.8 ± 1.8 mm. At the 9-month follow-up, the overall membrane elevation was slightly reduced to 13.0 ± 1.6 mm (*p* = 0.000), with a mean bone gain of 9.6 ± 2.4 mm. The amount of bone above the implant tip was 3.4 ± 1.7 mm at placement, decreasing to 3.0 ± 1.2 mm at follow-up (*p* = 0.007). **Conclusions**: The CAS kit technique combined with tissue-level implants demonstrated significant vertical bone gain and high implant survival rates without complications. This minimally invasive approach proved effective and safe for sinus augmentation in patients with limited residual bone height. The findings support the CAS kit’s potential as a preferred technique for maxillary sinus elevation. Further research with larger cohorts and long-term follow-up is needed to validate these results.

## 1. Introduction

Sinus augmentation procedures have been widely utilized for over 50 years, first described by Tatum Jr. in 1986 [1]. These procedures are crucial in dental implantology, particularly for patients with insufficient vertical bone height in the posterior maxilla, which is often due to maxillary sinus pneumatization and alveolar bone resorption. By increasing the available bone volume, sinus augmentation facilitates the placement of dental implants with enhanced stability and long-term success rates [2,3]. Two primary techniques are employed for maxillary sinus membrane elevation: the lateral window approach and the alveolar crestal approach. The lateral window technique, originally developed by Tatum, involves creating a bone window on the lateral wall of the maxillary sinus to enable the elevation of the Schneiderian membrane [4]. This approach allows for the placement of a bone graft beneath the elevated membrane, promoting new bone formation and facilitating implant stability. Despite its effectiveness, the lateral approach is associated with potential complications such as membrane perforation, postoperative discomfort, and longer recovery times [5]. In contrast, the crestal approach to sinus elevation, first introduced by Summers in 1994 [6] utilizes an osteotome technique that accesses the sinus floor through the alveolar crest. This less invasive method relies on controlled fractures of the sinus floor to achieve membrane elevation, often using bone graft materials to enhance bone regeneration. Over the years, multiple modifications to the crestal technique have been proposed, incorporating piezoelectric devices, balloon-assisted elevation, and hydraulic pressure systems to minimize trauma and improve surgical outcomes [7,8,9,10]. A recent randomized controlled trial by Xhanari et al. [7] compared the lateral and crestal techniques, demonstrating successful outcomes with both methods. Notably, the crestal approach required less surgical time and was preferred by patients due to its minimally invasive nature and reduced postoperative discomfort. These findings underscore the growing preference for less invasive techniques that enhance patient experience without compromising clinical success. Further advancements in sinus augmentation were introduced by Better et al. [8] and subsequently by Tallarico et al. [9,10] who described a minimally invasive staged approach utilizing a specialized dental implant. This method involves the use of hydraulic pressure to elevate the Schneiderian membrane while simultaneously placing a flowable bone substitute. This innovative approach minimizes the risk of membrane perforation and reduces surgical complexity. However, the necessity for dedicated implants and the technical learning curve remain limitations of this technique. Recent innovations in hydraulic sinus lifting were explored by Yassin Alsabbagh et al. [11] who compared an inflatable balloon technique and the crestal approach sinus (CAS kit) with conventional sinus floor elevation methods. Their findings revealed that both the balloon and CAS kit techniques were superior to traditional bone-added osteotome sinus floor elevation, offering improved safety and efficacy [11]. This preliminary data were confirmed with a series of manuscripts validating the CAS kit technique in combination with bone level implants [12,13,14]. These advancements reflect a continuous evolution in sinus augmentation techniques, driven by the need for less invasive, more predictable procedures that enhance patient comfort and clinical outcomes.

The crestal approach sinus lift is commonly used when only a moderate augmentation of the sinus floor (minimum 3 mm) is required [15]. Contraindications include severe sinus pathology (e.g., chronic sinusitis), insufficient residual bone (less then 3 mm), uncontrolled systemic diseases (e.g., diabetes), active periodontal disease, or poor oral hygiene [16]. Additionally, patients with a history of sinus surgery or anatomical variations such as a thin sinus membrane may not be suitable candidates for this procedure [17].

Despite significant progress, challenges such as membrane perforation, graft stability, and the long-term success of implants in augmented sinuses remain areas of active research. Ongoing studies are focused on optimizing graft materials, refining surgical techniques, and developing innovative tools to further improve the safety and effectiveness of sinus augmentation procedures. As the field continues to evolve, future research should aim to establish standardized protocols and long-term clinical data to support evidence-based practice in maxillary sinus augmentation.

The aim of this retrospective study is to evaluate the increase in vertical bone level, measured in millimeters, following sinus lift procedures using the CAS kit technique in combination with a tissue-level implant. Additionally, the quantity of bone between the apex of the implant and the Schneider membrane will be assessed. The following manuscript was written according to the Strengthening the Reporting of Observational Studies in Epidemiology (STROBE) Statement: guidelines for reporting observational studies.

## 2. Material & Methods

### 2.1. Study Design and Ethical Principles

This study was designed as a retrospective case series study and conducted in Rome, at the “San Pietro Hospital, Fatebenefratelli”. All the patients were informed about the nature of the treatment, and their written consent was obtained. Data collection was designed to preserve patient anonymity. “Due to this paper is a case series in which the best possible treatment was delivered, and no prospective research was carried out, in accordance with European and international guidelines (see below) no ethical concern was present, so no ethical approval was requested (Directive 2001/20/EC of the European Parliament and of the Council of 4 April 2001 on the approximation of the laws, regulations and administrative provisions of the member states relating to the implementation of good clinical practice in the conduct of clinical trials on medicinal products for human use) [18]. Moreover, any Institutional Review Board approval was not required for this case series, since we did not test any experimental technique, in accordance with relevant legislation (Artt. 10 e 320 cod.civ. and artt. 96 e 97 legge 22.4.1941, n. 633). All the surgical and prosthetic procedures were performed by two expert clinicians (FMC and AM) in accordance to the ethical principles reported in the Declaration of Helsinki, regarding human experimentation developed originally in 1964 and amended at the General Assembly in October 2024.

### 2.2. Inclusion and Exclusion Criteria

The present retrospective study evaluated data collected from any partially edentulous patient, aged 18 years or older, rehabilitated in the posterior maxilla (premolars and molars) with a tissue level implant in combination with sinus lift by a crestal approach, with a minimum residual bone height (RBH) of 2 mm, between September 2021 and October 2024. Exclusion criteria were the following: symptomatic sinus membrane pathology and/or obstruction of the maxillary ostium; immediate implants (at least 4 months after tooth extraction); heavy smoking (≥10 cigarettes/day); untreated periodontitis; poor oral hygiene and motivation (full-mouth bleeding on probing and full-mouth plaque index 25%); general medical contraindication to oral surgery (American Society of Anesthesiologists class III or IV); irradiation in the head and neck area less than 5 years before implantation; psychiatric problems; alcohol or drug abuse; pregnancy or nursing; any interfering medication (steroid or bisphosphonate therapy).

### 2.3. Surgical and Prosthetic Procedures

Cone-beam computed tomography (CBCT) scans were used for initial screening. After local anesthesia, a full-thickness flap was elevated to expose the alveolar ridge. A drilling sequence was performed using the CAS kit system (Osstem Implant CO., LTD., Seoul, Republic of Korea), according to the manufacturer’s instructions. Step-by-step technique is reported:

- Cortical bone marking was performed to display drilling location, using the guide drill (2.0/2.7 mm drill) in combination with a 2 mm stopper, at 1.000 to 1.500 rpm.
- Twist drill of 2.2 mm was used in combination with stopper (1 mm shorten than the working length), at 1.000 to 1.500 rpm. Working length was defined as the distance between the bone crest and the sinus floor, at the intended implant placement, along the long implant axis. Twist drill of 2.2 mm was not used in case with 2 and 3 mm of RBH.
- CAS drills (Osstem Implant CO., LTD., Seoul, Republic of Korea) with rounded edges to prevent damage to the Schneiderian membrane were used with stoppers to approach the maxillary sinus at 400 to 800 rpm, according to the recommended protocol [14].
- A hydraulic membrane lifter was used to infuse, slowly and gradually, sterile saline to separate the membrane, according to the differences between the planned implant length and the residual bone height (lift height, Table 1). The injection and retrieval of saline, at steps of 0.5 cc, from 1.0 to 1.5 maximum, was used.

- CAS drills were used to enlarge the osteotomy site according to the final diameter of the planned implants and the bone density (2.8 mm drill for 3.8 mm diameter implants and 3.1 diameter drill for 4.25 mm diameter implants), and gain access to the sinus for bone grafting. A bone carrier was used to graft the sinus at the intended implant site, using an anorganic bovine bone material (Bio-Oss, granule sizes 0.25 to 1 mm, Geistlich Pharma AG, Wolhusen, Switzerland). The amount of bone graft was decided according to the lift height (Table 1).
- Finally, a self-tapping implant (PRAMA, Sweden and Martina) was placed. This implant was characterized by a specific and unique 2.8 mm convergent neck with a microtextured surface (UTM surface) and Zirconium Titanium (ZirTi) implant body surface. The neck design was developed to adopt the biologically oriented preparation technique (BOPT). All the implants were installed with a minimum seating torque of 25 Ncm. After that, a healing abutment was immediately connected in all the cases, and the flap sutured without any tension. Patients received post-intervention instructions, pain killers (Ibuprofen 600 milligrams as needed, maximum three times a day) and antibiotics for six days (1 g Amoxicillin twice per day for six days). Six months after the implant placement, a conventional impression was taken in all the patients, and, 3 to 4 weeks later, single, screw-retained porcelain fused to metal crowns was delivered. Patients were enrolled in an hygiene maintenance protocol. At each appointment, the restoration was checked and a radiographic control was carried out.

### 2.4. Outcome Measures

- Primary outcome measures were implant and prosthetic survival rates, and any biological and technical complications. Implant failure was defined as implant mobility or any infection dictating implant removal, implant fracture, or any other mechanical complication rendering the implant useless, while a prosthesis was considered a failure if it needed to be replaced by a new prosthesis. Any biologic (pain, swelling, mobility, and suppuration) or technical complication (abutment or veneering material fracture, screw loosening or fracture) was recorded during follow-up.
- Secondary outcomes measures were the overall tend effect (residual bone height + implant + graft) evaluated immediately after implant placement, and the final overall height of the elevated sinus evaluated at the crown delivery, 9 months after implant placement. All the post-operative measures were taken on the panoramic radiography.

### 2.5. Statistical Analysis

Data were collected in the Numbers software for MacOS Sequoia (Version 14.2). All data analysis was carried out according to a pre-established analysis plan by a biostatistician with expertise in dentistry. A descriptive analysis was performed using mean ± SD, median, and 95% confidence interval (CI). Differences in means between time points were compared by non-parametric Wilcoxon signed-rank test. The implant was the statistical unit of the analyses. All statistical comparisons were conducted at the 0.05 level of significance.

## 3. Results

**Figure 1:**
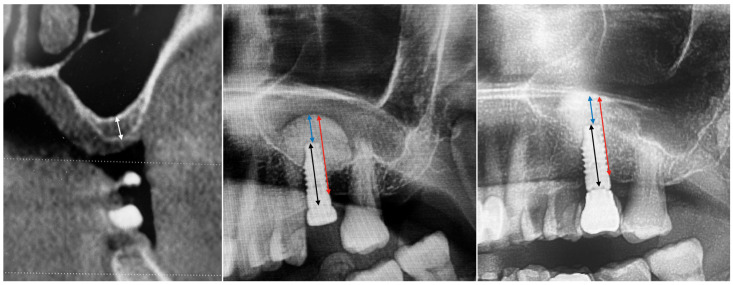
From **left** to **right**: preclinical situation, implant placement and sinus lift, nine months after implant placement. White line: residual bone height (RBH); black line: implant length; red line: overall amount of bone (OAB); blue line: bone above the implant tip (BAI).

The present retrospective study evaluated data collected from any partially edentulous patient, aged 18 years or older, rehabilitated in the posterior maxilla (premolars and molars) with at least one tissue level implant in combination with sinus lift by a crestal approach, with a minimum residual bone height (RBH) of 2 mm, between September 2021 and October 2024. Data from 36 potential candidates were initially included. Of these, only 15 patients with 20 implants met the inclusion/exclusion criteria. Of the 21 excluded patients, 16 did not have the panoramic radiography at the crown delivery. Three patients did not receive the final crown due these patients moved in an other city. Two patients had serious health problems (one had an heart attack and one a stroke). All the included patients had a radiographic control nine months after implant placement. The mean follow-up was 19.5 ± 6.4 months (from 9 to 30). None of the implants or prostheses failed, and no technical or biological complications were experienced. The mean residual bone height (RBH) at implant placement was 4.2 ± 1.4 mm (from 2.0 to 6.7 mm). The overall membrane elevation was 13.8 ± 1.8 mm (from 10.9 to 17.8). The difference baseline (RBH) was 9.6 ± 2.4 mm (from 5.7 to 14.3; *p* = 0.000). The amount of bone above the implant tip was 3.4 ± 1.7 mm (from 1.3 to 7.9). At the nine-month follow-up, the overall membrane elevation was 13.0 ± 1.6 mm (from 10.4 to 16.1). The difference comparing with implant placement was 0.8 ± 0.7 mm (from 0.0 to 2.6; *p* = 0.000). The amount of bone above the implant tip was 3.0 ± 1.2 mm (from 1.3 to 5.7 mm). The difference comparing with implant placement was 0.4 ± 0.6 mm (from 0.0 to 2.3; *p* = 0.007). Data and an explanatory case are reported in Figure 1 and Table 2(a), (b).

## 4. Discussion

This retrospective study evaluated the effectiveness of the CAS kit technique combined with tissue-level implants for sinus lift procedures in the posterior maxilla. The findings demonstrated a significant increase in vertical bone height, with a mean membrane elevation of 13.8 ± 1.8 mm at implant placement, which slightly decreased to 13.0 ± 1.6 mm at the 9-month follow-up. Importantly, no implant or prosthetic failures were observed, and no biological or technical complications were reported, highlighting the safety and reliability of this approach.

These results are consistent with previous studies on minimally invasive sinus augmentation techniques. For instance, Yassin Alsabbagh et al. [11] reported improved safety and efficacy with the CAS kit compared to traditional osteotome sinus floor elevation, attributing these outcomes to the controlled hydraulic membrane elevation that reduces trauma to the Schneiderian membrane. The present study corroborates these findings, demonstrating that the CAS kit technique effectively minimizes complications while achieving substantial vertical bone gain. Additionally, the use of tissue-level implants may have contributed to the favorable outcomes by enhancing soft tissue stability and reducing peri-implantitis risk [19].

When compared to the lateral window approach, which is associated with higher complication rates, including membrane perforation and postoperative discomfort, the crestal approach using the CAS kit offers significant advantages. In line with the randomized controlled trial by Xhanari et al. [7] which highlighted shorter surgical time and better patient acceptance with the crestal approach, this study further supports the growing preference for less invasive techniques. The CAS kit’s hydraulic pressure system facilitates a gradual and controlled elevation of the Schneiderian membrane, reducing the risk of perforation and enhancing graft stability. In this approach, a set of rounded drills with metal stoppers, an hydraulic lifter, and a bone graft carrier, were safely used. The procedure begins with a 2-mm twist drill, followed by the use of drills to deepen the osteotomy. The sinus floor is then perforated with a 2.8 mm bur, without breaching the membrane. A depth gauge ensures membrane integrity, and the hydraulic lifter is inserted and stabilized. Saline solution is injected, with 0.50 mL elevating the membrane by up to 3 mm [10]. This process is repeated until the desired elevation is achieved.

The choice to graft or not the sinus is widely discussed in the literature. According to a recent systematic review by Silva et al. [20], maxillary sinus lift surgery, with or without graft material, is a safe procedure with a low complication rate and predictable results. According to Gatti et al. [14] grafting the sinus with inorganic bovine bone allow to reduce the graft remodeling, enacting the tend effect during osseointegration, and allowing a radiographic control. For the latter, in the present research, all the cases were grafted independently by the residual bone height.

This study demonstrated that the CAS kit technique combined with tissue-level implants is an effective and safe approach for sinus lift procedures in the posterior maxilla. The technique resulted in significant vertical bone gain, maintained stability over a nine-month follow-up, and showed high implant and prosthetic survival rates with no biological or technical complications. These findings highlight the potential of the CAS kit technique to become a preferred option for maxillary sinus augmentation, particularly for patients with limited residual bone height.

The minimally invasive nature of the CAS kit technique, combined with its ability to minimize membrane perforation risks and postoperative discomfort, makes it a valuable alternative to conventional lateral window approaches. Despite its advantages, the crestal approach to sinus is not without potential risks and complications. One of the primary concerns is the risk of Schneiderian membrane perforation, which can compromise the outcome of the graft and increase the likelihood of postoperative sinusitis or infection. Limited visibility and tactile feedback inherent in the crestal approach may further increase this risk, particularly in cases with a thin or irregular sinus floor. Further research is required to validate these outcomes in larger, multicenter studies with long-term follow-up. Future investigations should also explore the comparative effectiveness of the CAS kit with other emerging sinus lift techniques and evaluate the long-term success of implants placed in augmented sinuses [21,22,23,24].

Despite these promising results, several limitations should be acknowledged. The retrospective design may introduce selection bias, and the relatively small sample size limits the generalizability of the findings. Additionally, the reliance on panoramic radiography, rather than cone-beam computed tomography, may have affected the precision of vertical bone height measurements. Future studies should employ CBCT for more accurate assessments and include a control group to compare the CAS kit technique with other sinus lift methods.

The CAS kit system represents a promising advancement in sinus augmentation, contributing to enhanced patient comfort, reduced surgical complexity, and reliable clinical outcomes. Furthermore, the long-term stability of the augmented bone and the survival rate of implants beyond the nine-month follow-up period remain to be established. As the field continues to evolve, standardized protocols and robust clinical data are essential for optimizing maxillary sinus augmentation practices. Prospective randomized controlled trials with larger cohorts and extended observation periods are necessary to validate these preliminary findings. Comparative studies with other minimally invasive techniques, such as balloon-assisted or piezoelectric sinus elevation, could provide valuable insights into the optimal approach for different clinical scenarios.

## 5. Conclusions

The CAS kit system combined with tissue-level implants demonstrated significant vertical bone gain and high implant survival rates without complications, providing effective and safe sinus augmentation in patients with limited residual bone height. Further research with larger cohorts and long-term follow-up is needed to validate these results.

## Figures and Tables

**Table 1:** Amount of bone graft and saline according to the sinus anatomy.

Lift Height (mm)	Bone Graft (cc)	Saline (cc)
3	0.36	0.5–1
4	0.5	1
5	0.7	1–1.5
6	0.9	1.5–2

**Table 2:** (**a**) Radiographic outcome measures in mm. (**b**) Descriptive statistics.

(a)											
Implants’ Characteristics				Implant Placement				9 Months Follow-Up			
Case N°	Site	Diameter (mm)	Length (mm)	RBH (mm)	OAB (mm)	Difference (mm)	BAI (mm)	OAB (mm)	Difference (mm)	BAI (mm)	Last FU (Months)
1	26	4.25	10	4.6	13.66	9.06	2.31	13.21	0.45	2.13	9
2	15	4.25	10	3.29	13.99	10.7	3.10	13.14	0.85	3.05	11
3	25	3.8	10	4.94	13.90	8.96	2.69	13.84	0.06	2.7	11
4	26	4.25	10	3.38	13.29	9.91	2.73	12.34	0.95	2.45	11
5	25	3.8	10	6.6	14.84	8.24	3.04	13.44	1.4	2.78	14
6	16	4.25	10	3.28	11.8	8.52	1.37	11.05	0.75	1.28	15
7	26	4.25	10	4.27	14.25	9.98	4.42	13.65	0.6	3.45	16
8	15	4.25	10	4.72	13.02	8.3	3.04	12.25	0.77	2.89	16
9	26	4.25	10	2.43	16.69	14.26	6.14	14.11	2.58	4.15	19
10	26	4.25	10	4.88	11.08	6.2	1.61	10.78	0.3	1.45	19
11	25	3.8	10	6.17	15.67	9.5	4.11	15.60	0.07	3.98	21
12	26	3.8	10	3.66	15.45	11.79	4.49	15.03	0.42	3.90	21
13	16	4.25	10	3.78	17.77	13.99	7.94	16.09	1.68	5.66	22
14	17	4.25	8,5	2.45	13.81	11.36	4.44	13.13	0.68	4.15	22
15	15	4.25	10	4.57	15.19	10.62	3.56	14.67	0.52	3.11	24
16	27	4.25	10	2.0	12.03	10.03	2.36	11.42	0.61	2.09	25
17	16	3.8	10	4.11	10.87	6.76	1.46	10.37	0.5	1.44	27
18	17	3.8	10	5.59	11.27	5.68	1.31	11.17	0.1	1.35	27
19	26	4.25	10	6.72	13.24	6.52	3.89	13.20	0.04	3.77	29
20	16	4.25	10	2.0	13.97	11.97	4.53	12.02	1.95	4.23	30
(**b**)											
				**Implant Placement**				**9 Months Follow-Up**			
**Implants (n = 20)**				**RBH (mm)**	**OAB (mm)**	**Difference (mm)**	**BAI (mm)**	**OAB (mm)**	**Difference (mm)**	**BAI (mm)**	**Last FU (Months)**
Mean (mm) and SD (mm)				4.2 (1.4)	13.8 (1.8)	9.6 (2.4)	3.4 (1.7)	13.0 (1.6)	0.8 (0.7)	3 (1.2)	19 (6)
Confidence interval (mm)				0.6	0.8	1	0.7	0.7	0.3	0.5	2.8
Min (mm)/Max (mm)				2.0–6.7	10.9–17.8	5.7–14.3	1.3–7.9	10.4–16.1	0.0–2.6	1.3–5.7	9–30
*p* Value						0.000			0.000		

RBH = residual bone height; OAB = overall amount of bone; BAI = bone above the implant tip; FU = Follow-up; SD = standard deviation.

## Data Availability

The original contributions presented in this study are included in the article. Further inquiries can be directed to the corresponding author.

## References

[1] Tatum H. (1986). Maxillary and sinus implant reconstructions. Dent. Clin. N. Am..

[2] Sirinirund B., Rodriguez Betancourt A.B., Scaini R., Wu Y.C., Chan H.L. (2025). Minimally Invasive Sinus Augmentation: A Systematic Review. Clin. Implant. Dent. Relat. Res..

[3] Alshamrani A.M., Mubarki M., Alsager A.S., Alsharif H.K., AlHumaidan S.A., Al-Omar A. (2023). Maxillary Sinus Lift Procedures: An Overview of Current Techniques, Presurgical Evaluation, and Complications. Cureus.

[4] Alsharekh M.S., Almutairi A.A., Jahlan A.S., Alhazani A.S., Almohaimeed S.M., Aljnoubi L.A., AlGhamdi G.A., AlBenyan T.T., Alduhyaman S.F., Alnaffaie N.M. (2024). Evolving Techniques and Trends in Maxillary Sinus Lift Procedures in Implant Dentistry: A Review of Contemporary Advances. Cureus.

[5] Molina A., Sanz-Sánchez I., Sanz-Martín I., Ortiz-Vigón A., Sanz M. (2022). Complications in sinus lifting procedures: Classification and management. Periodontology.

[6] Summers R.B. (1994). A new concept in maxillary implant surgery: The osteotome technique. Compendium.

[7] Xhanari E., Tallarico M., Meloni S.M., Kalemaj Z., Ceruso F.M., Dedaj E. (2019). Crestal versus lateral sinus lift: One year result from a within-patient randomized controlled trial. Clin. Trials Dent..

[8] Better H., Slavescu D., Barbu H., Cochran D.L., Chaushu G. (2014). Minimally invasive sinus lift implant device: A multicenter safety and efficacy trial preliminary results. Clin. Implant. Dent. Relat. Res..

[9] Decker A.M., Stuhr S., Testori T., Wang H. (2024). Clinical and radiographic changes following transcrestal sinus augmentation: A scoping review of the last 25 years. Clin. Implant. Dent. Relat. Res..

[10] Tallarico M., Cochran D.L., Xhanari E., Dellavia C., Canciani E., Mijiritsky E., Meloni S.M. (2017). Crestal sinus lift using an implant with an internal L-shaped channel: 1-year after loading results from a prospective cohort study. Eur. J. Oral Implantol..

[11] Yassin Alsabbagh A., Alsabbagh M.M., Darjazini Nahas B., Rajih S. (2017). Comparison of three different methods of internal sinus lifting for elevation heights of 7 mm: An ex vivo study. Int. J. Implant. Dent..

[12] Wu H., Wang J., Wang C., Yang X., Gong Q., Su W., Cheng A., Fan Y. (2022). A Comparison of Elevation, Perforation Rate, and Time Spent for the Crestal Sinus Elevation Intervened by Piezosurgery, CAS-Kit, and Osteotome in a Novel Goat Model. J. Maxillofac. Oral Surg..

[13] Kim Y.K., Cho Y.S., Yun P.Y. (2013). Assessment of dentists’ subjective satisfaction with a newly developed device for maxillary sinus membrane elevation by the crestal approach. J. Periodontal Implant. Sci..

[14] Gatti F., Gatti C., Tallarico M., Tommasato G., Meloni S.M., Chiapasco M. (2018). Maxillary Sinus Membrane Elevation Using a Special Drilling System and Hydraulic Pressure: A 2-Year Prospective Cohort Study. Int. J. Periodontics Restor. Dent..

[15] Jensen O.T., Shulman L.B., Block M.S., Iacono V.J. (1998). Report of the Sinus Consensus Conference of 1996. Int. J. Oral Maxillofac. Implant..

[16] Pjetursson B.E., Tan W.C., Zwahlen M., Lang N.P. (2008). A systematic review of the success of sinus floor elevation and survival of implants inserted in combination with sinus floor elevation. J. Clin. Periodontol..

[17] Wallace S.S., Froum S.J. (2003). Effect of maxillary sinus augmentation on the survival of endosseous dental implants. A systematic review. Ann. Periodontol..

[18] European Commission—European Medicines Agency Report on the conference on the Operation of the Clinical Trials Directive (Directive 2001/20/EC) and Perspectives for the Future, Conference held on 3 October 2007 at the EMEA, London (Report Issued on November 30, 2007; Doc. ref.: EMEA/565466/2007). https://health.ec.europa.eu/system/files/2017-02/ec_emea_conference_on_clinical%252520_trials_en_0.pdf.

[19] Ceruso F.M., Ieria I., Tallarico M., Meloni S.M., Lumbau A.I., Mastroianni A., Zotti A., Gargari M. (2022). Comparison between Early Loaded Single Implants with Internal Conical Connection or Implants with Transmucosal Neck Design: A Non-Randomized Controlled Trial with 1-Year Clinical, Aesthetics, and Radiographic Evaluation. Materials.

[20] Silva L.D., de Lima V.N., Faverani L.P., de Mendonça M.R., Okamoto R., Pellizzer E.P. (2016). Maxillary sinus lift surgery-with or without graft material? A systematic review. Int. J. Oral Maxillofac. Surg..

[21] Speroni S., Bosco F., Ferrini F., Pittari L., Nota A., Tecco S. (2024). The Use of a Surgical Template for the Insertion of Dental Implants and Sinus Lift with the Summers Technique Based on Digital Planning: A Case Report. Prosthesis.

[22] Kondratiev A., Demenko V., Linetskiy I., Weisskircher H.-W., Linetska L. (2024). Evaluation of Bone Turnover around Short Finned Implants in Atrophic Posterior Maxilla: A Finite Element Study. Prosthesis.

[23] Comuzzi L., Romasco T., Piattelli A., Inchingolo F., Mourão C.F., Di Pietro N. (2024). Comparative Evaluation of Primary Stability in Truncated Cone Implants with Different Macro-Geometries in Low-Density Polyurethane Blocks Simulating Maxillary Sinus Rehabilitations. Prosthesis.

[24] Chan H.L., Oh T.J., Fu J.H., Benavides E., Avila-Ortiz G., Wang H.L. (2013). Sinus augmentation via transcrestal approach: A comparison between the balloon and osteotome technique in a cadaver study. Clin. Oral Implant. Res..

